# Improving Course Evaluation Response Rates: A Success Story

**DOI:** 10.1007/s40670-025-02464-y

**Published:** 2025-07-22

**Authors:** Jacqueline L. Gauer, Stephen J. Martin, Eric C. Martin LaPlant

**Affiliations:** University of Minnesota Medical School, 420 Delaware St SE, MMC 293, Minneapolis, MN 55455 USA

**Keywords:** Course evaluations, Continuous quality improvement, Program evaluation, Response rates, Curricular review, Evaluation of teaching

## Abstract

Student participation in course evaluations is essential for curriculum improvement, but response rates have been declining at many institutions. After other strategies failed, the University of Minnesota Medical School made completing course evaluations a required professional expectation. This policy was developed through a system-level approach where input was solicited from many different stakeholders, including students. It is reinforced by student buy-in, clear communication, providing students with dedicated time for completing evaluations and the ability to opt out of completing any particular evaluation, and non-punitive follow-up meetings for non-compliant students. Response rates rose from about 50 to 94% in the first semester after implementation, with minimal pushback from students. This approach offers a scalable model for institutions seeking to improve response rates, ensuring more representative feedback to guide curriculum development.

## Background

Low student participation in course evaluations poses a significant challenge, as regular and comprehensive student feedback is essential for continuous curriculum improvement [1, 2]. Course evaluations can provide vital insights into the effectiveness of teaching methods, course content, and overall student satisfaction [3]. High response rates ensure that the feedback collected is representative of the entire student body, which helps provide a comprehensive and accurate picture of course effectiveness and areas needing improvement. However, numerous institutions have reported low response rates, especially with the transition from traditional paper-based evaluations to online methods. While online evaluations offer advantages such as administrative efficiency and more detailed feedback, they often result in lower participation rates [4, 5].

Several factors contribute to low response rates. Students often perceive that their feedback does not lead to tangible changes, which diminishes their willingness to participate in evaluations. According to self-determination theory, students are more likely to complete evaluations when they perceive autonomy, competence, and relatedness [6]. Additionally, students are more willing to participate when they believe their input is valued and leads to meaningful improvements [7]. Furthermore, faculty perspectives on evaluations can affect student participation; if faculty do not emphasize the importance of evaluations or follow up on feedback, students may not see the value in completing them [5]. Another major issue is survey fatigue. When students are overwhelmed by the frequency and volume of surveys, participation rates decline, and feedback may become biased, as only the most motivated students are likely to respond [8].

Despite recognizing the problem and various efforts to address it, challenges remain in achieving consistently high course evaluation response rates. Strategies such as providing incentives like extra credit or early access to grades have been attempted to encourage participation [5]. However, the over-reliance on incentives fails to address deeper issues such as survey fatigue and the perceived irrelevance of the feedback process. Non-incentive strategies, such as fostering a respectful classroom environment and clearly communicating the value and impact of student feedback, tend to be more effective in the long run [9]. Yet, without consistent follow-through and support from faculty, these strategies can also fall short. These findings highlight the need for more comprehensive and sustainable approaches that address the root causes of low response rates rather than relying on incentives.

Until recently, this issue was particularly pronounced at the University of Minnesota Medical School (UMMS). By academic year (AY) 2020–2021, average course evaluation response rates had declined to 45% for the Twin Cities campus and 56% for the Duluth campus, and the downward trend was expected to continue. These low rates compromised the quality and reliability of the feedback collected, making it difficult to implement meaningful curriculum changes [4]. Course directors, for example, struggled to determine whether their course evaluations were representative, leading to reluctance in making changes that may have been desired only by the most vocal students. Over the years, faculty and staff attempted many of the strategies commonly recommended in the literature for improving response rates, such as sending students multiple reminders and communicating the importance of feedback with the students [4]. Unfortunately, these efforts were unable to reverse or even slow the decline in response rates. Therefore, it was determined that a more systematic approach to addressing the problem would be required.

## Activity

In February 2021, the institution formed a Course Evaluation Response Rate Task Force (CERRTF). CERRTF included representation from staff, faculty, and students. Based on literature reviews, consultations with stakeholders, and their own experiences, the group brainstormed multiple potential strategies for improving response rates. Ultimately, CERRTF proposed a 14-step action plan (Table 1) for implementation in AY 2021–2022, setting response rate goals of 60% (threshold), 70% (target), and 80% (stretch) to evaluate success. These benchmarks were based on published literature indicating that response rates in the 60–70% range are generally considered acceptable for drawing meaningful conclusions from course evaluation data [4].

**Table 1:** Original recommendations of the University of Minnesota’s Course Evaluation Response Rate Task Force, which were implemented for Academic Year 2021–2022

Timing	Recommendation
Before evaluations are delivered	Include information during Orientation for incoming first-year students regarding the importance of completing course evaluations
	Associate Dean for Undergraduate Medical Education (UME) and Duluth Campus Dean email students at the beginning of the academic year and/or at the end of each term to encourage them to complete their evaluations
	When UME Evaluation and Curriculum staff meet with course leads and/or faculty, include discussions of how the faculty can encourage course evaluation completion
	Course Directors to include information in their introductory course sections about changes that have been made to their courses based on previous evaluation feedback (a PowerPoint slide will be provided to facilitate this)
	When communicating regarding evaluations, emphasize the unique role of course evaluations. Inform students that evaluations are used in promotion and tenure. Communicate that we need all feedback—positive, constructive, and neutral. Emphasize that this is a professional responsibility
	Offer Course Directors the option of having their previous year’s Annual Course Review posted to their Canvas page
	Change the delivery date of course evaluations to have them open before the final exam
During the evaluation fielding period	Course Directors to discuss the importance of completing evaluations during their last session and/or review session (a PowerPoint slide will be provided to facilitate this)
	When possible, Course Directors to schedule time at the end of their course for students to complete evaluations
	Course Directors/Managers to set evaluation reminder announcements in Canvas
	Shorten evaluation forms to the extent possible
	Update the evaluation delivery and reminder emails to have either the Associate Dean for Undergraduate Medical Education (UME) or Duluth Campus Dean as the “from” address, depending on the student’s campus
	Add clarifications to course evaluation forms indicating which sections are required
After fielding period	At Course Debriefs, Curriculum Directors to encourage Course Directors and/or Leads to email a follow-up to the class with a summary of what was discussed and any commitments made in response to evaluation feedback

CERRTF regrouped in February 2022 to review the outcomes of the action plan, which were not promising. Response rates for the Fall courses had improved by only 5% for first-year students and continued to decline for second-year students. These results did not meet the task force’s goals and required significant effort from staff and faculty. In response, CERRTF resumed meetings and expanded its consultative efforts, engaging the Student Council, conducting student surveys, and reaching out to other health science programs at the University, as well as the national DR-ED medical education listserv. It became increasingly clear that the simplest and most effective approach would be to make completing course evaluations a required professional expectation of the medical students. Additionally, much of the student body appeared to support this change.

As could be expected, both staff and faculty had reservations about the requirement. Concerns included potential student burnout, declines in overall satisfaction, and the risk of disengaged or frustrated students providing inaccurate data. To mitigate these concerns, CERRTF proposed additional strategies that would be used to support the implementation of the requirement, including allocating dedicated time for evaluations in student calendars and emphasizing the importance of feedback during Orientation, and incorporating some of the changes made previously such as reducing overall survey load to the extent possible and communicating with students about the importance of providing feedback during Orientation (Table 2).

**Table 2:** Second set of recommendations of the University of Minnesota’s Course Evaluation Response Rate Task Force, which were implemented for Academic Year 2022–2023

Primary recommendation	The Course Evaluation Response Rate Task Force (CERRTF) recommends that completing preclinical course evaluations be considered a required professional expectation starting in AY 2022–2023 To align with University of Minnesota policy, each course evaluation will include an “Opt Out” button, which students can select if they wish to opt out of providing the information requested for that particular evaluation. Students who elect to opt out of an evaluation will be considered to have met the requirement, but their evaluation will not be considered completed when calculating the response rate for the course
Recommended additional supporting actions	We recommend that time be added to curriculum calendars for students to complete their course evaluation(s)
	Whenever possible, we ask that Course Directors communicate with students regarding the feedback provided by their predecessors, as well as how that feedback was used to improve the course
	Whenever possible, we ask that Course Directors communicate with students regarding the feedback received from their own class, along with any changes they intend to make for future courses
	We will be distributing materials during Orientation on both campuses, communicating the requirement and providing training on how students can provide useful, actionable feedback
	We recommend putting the requirement in the context of professionalism, and that it be accompanied by a more general explanation of the expectations for professional responsibilities and behavior by medical students
	We will provide additional materials for all students explaining the various ways they can provide feedback and the unique purposes of each
	We recommend reducing the overall number of surveys being distributed to students, where possible
	If a student does not complete a course evaluation, we recommend the requirement be emphasized via a method such as escalating communications from individuals from the Office of Medical Education

Ultimately, the recommendations were approved in June 2022 and implemented in Fall 2022. The requirement was enforced by asking non-compliant students to meet with the Assistant Dean for Assessment and Evaluation to review the requirement and discuss its importance (these meetings were meant to be informative rather than punitive). The institution tracks whether or not a student has submitted an evaluation, but the contents of students’ individual responses remain anonymous.

In order to align with University policy, avoid issues of coercion, and account for students who may be unable to complete an evaluation, each course evaluation includes an item asking the student if they wish to opt out of the evaluation. Students must open and submit the survey, but they can choose to opt out and still be considered in compliance. To have a more accurate picture of the representativeness of course evaluation data, the institution therefore tracks two numbers for each evaluation: (1) response rate: the total number of surveys submitted, divided by the number of students enrolled in the course; and (2) completion rate: the number of students who completed the survey and did not choose to opt out, divided by the number of students enrolled in the course.

## Results

The effects of the new course evaluation completion requirement were clear and immediate following its implementation. Response rates for course evaluations in Fall 2022 jumped to an average of 97% (range: 92–100%), a 49% increase from the previous Fall, far exceeding the task force’s 80% stretch goal. Completion rates averaged 94% (range: 91–98%) for that semester.

**Fig. 1:**
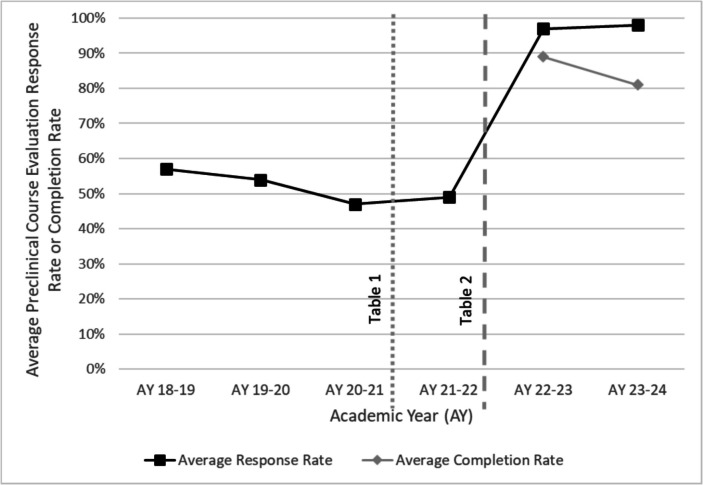
Average response rates (submitted evaluations/enrolled students) and completion rates ((submitted evaluations − opt-out option selected)/enrolled students) for student evaluations of preclinical courses, from Academic Year (AY) 2018–2019 through AY 2023–2024. The dotted vertical line indicates the implementation of the first set of task force recommendations (Table 1) at the beginning of AY 2021–2022; the dashed vertical line indicates the implementation of the second set of task force recommendations (Table 2), which included the recommendation to make completing course evaluations a required professional expectation of medical students, at the beginning of AY 2022–2023

With some variability, these improvements have been sustained over the 2 years since the requirement was implemented (Fig. 1).

Furthermore, the course evaluation requirement has not led to noticeable pushback from students. Staff responsible for administering evaluations and enforcing the requirement have received no direct complaints from students. Anecdotally, non-compliant students who meet with the Assistant Dean express their commitment to completing course evaluations in the future. Additionally, there has been no decline in the quality of either quantitative or qualitative feedback since the requirement was implemented.

## Discussion

The next step for the institution is to continue tracking response and completion rates to ensure they remain high. A potential limitation of the “opt-out” option is decreasing completion rates. The task force may be reformed if completion rates consistently fall below 70%. Potential solutions to mitigate a dropping completion rate include adjusting the professional expectation, incorporating sampling methods, or focusing more effort on changing the culture of evaluation. Thus far, completion rates have been sustained at levels above that cutoff.

This solution to the sticky problem of low course evaluation response rates could generalize well to other institutions. It was immediately effective at our large institution and does not require a large investment of time or resources to implement and sustain. While some institutions may hesitate to mandate evaluations due to concerns about student pushback, burnout, or low-quality responses, our institution offers a model to mitigate some of these concerns. Key features of our model include:

1. Incorporating student input in the development and implementation of the requirement;
2. Implementing supporting structures along with the requirement (such as dedicating time into students’ calendars and communicating with students during Orientation about why their feedback is so important);
3. Creating a flexible and non-punitive follow-up mechanism to enforce the requirement; and
4. Offering a means by which students can actively opt out from completing an individual evaluation as needed.

In summary, course evaluations provide important feedback that educational programs can use to improve their courses and curriculum. They are one of the most effective ways to ensure that student voices are represented in the institution’s evaluation processes. While previous literature has been reluctant to recommend making completing course evaluations a requirement [e.g., 4–12], our institution was able to implement such a requirement successfully and with minimal student pushback. While these initial results are promising, future research could help determine whether the changes are sustainable in the long term, whether the quality of feedback provided by students is impacted either positively or negatively by this policy, whether different cohorts of students perceive the policy in different ways (particularly as the students who were initially consulted in the development of the policy graduate), and the extent to which this approach is generalizable to other institutions. To that end, we encourage other institutions to explore this option and share their experiences.
